# An Easy Way to Solve the Stuck Leaflet Causing Aortic Regurgitation Following Transcatheter Aortic Valve Replacement

**DOI:** 10.1016/j.shj.2025.100697

**Published:** 2025-07-05

**Authors:** Neelima Katukuri, Brian R. Gebhardt, Matthew Lawlor, Jennifer Walker, Nikolaos Kakouros

**Affiliations:** a Division of Cardiovascular Medicine, University of Massachusetts, Worcester, Massachusetts, USA; b Division of Anesthesiology, University of Massachusetts, Worcester, Massachusetts, USA; c Division of Cardiothoracic Surgery, University of Massachusetts, Worcester, Massachusetts, USA

**Keywords:** Paravalvular regurgitation, Stuck leaflet, TAVR

## Abstract

• Intravalvular aortic regurgitation severity may be underestimated by transthoracic echocardiogram due to rapid pressure equalization.
• Transthoracic echocardiogram may not differentiate PVL from eccentric central aortic regurgitation; transesophageal echocardiogram may be required.
• Low diastolic pressures may be falsely attributed to vasoplegia or PVL.
• Mechanical resolution with catheter techniques may avoid valve-in-valve procedures.
• Rare cases of leaflet immobility are being reported even with newer generation SAPIEN 3 Ultra RESILIA valves.

A 91-year-old female with hypertension, hyperlipidemia, chronic kidney disease, coronary artery disease status post stent on aspirin and ticagrelor presented for dyspnea. Echocardiogram showed severe aortic stenosis with aortic valve area of 0.7 cm^2^ and left ventricular ejection fraction of 47%. The mean gradient was 59 mmHg. The patient Society of Thoracic Surgeons Risk Mortality was 22%, classifying her as very high risk.

A preprocedure imaging computed tomography scan showed severe peripheral arterial disease with calcification at common iliac artery and bilateral iliacs. The aortic valve and annulus were heavily calcified with area 402 cm^2^, perimeter of 73.6 mm, calcium score of 4494. Coronary heights were acceptable. The procedure was complicated by severe calcific stenosis of the right common iliac artery, which was treated using a 7-mm Shockwave balloon (Shockwave, Santa Clara, California). The right common femoral artery was used for large bore access and the valve crossed retrogradely in standard fashion, followed by placement of a SAFARI wire (Boston Scientific, Marlborough, Massachusetts) in the left ventricle. The 14-Fr sheath was removed, and the 27-mm Navitor valve (Abbott, Plymouth, Minnesota) was advanced using the inline sheath in a sheathless manner. However, the valve could not cross the mid-right common iliac artery due to buckling of the sheath in the poststenotic dilated segment. In view of this,^1^, ^2^, ^3^ we switched to an Edwards system. An Edwards e Sheath was placed with difficulty, facilitated by coating with Viper slide through which a 23-mm Edwards SAPIEN 3 Ultra RESILIA valve (Edwards, Irving, California) was deployed. Post implantation intraprocedural transesophageal echocardiogram demonstrated aortic regurgitation (AR) that persisted after postdilation using the delivery balloon and an additional 1 mL of volume. Further postdilation was avoided given concern for possible annular rupture. Notably, however, diastolic pressures were low (20-30 mmHg), necessitating the use of vasopressors. Over the following days, the patient could not be weaned from the latter, raising concerns that the AR was hemodynamically significant. In view of this, we planned to return to the cardiac catheterization lab for presumed paravalvular closure, a week after the initial implant.

Patient persisted to have low diastolic pressures, intraprocedural transesophageal echocardiogram showed severe AR occurred due to frozen leaflet (Figures 1 and 2). An innovative and cost-effective solution was chosen, where in a JR4 (Judkins Right) catheter with J wire was introduced to the base of the leaflet enabling central translocation of the leaflet resulting in resolution of AR (Figures 3 and 4).

**Figure 1:**
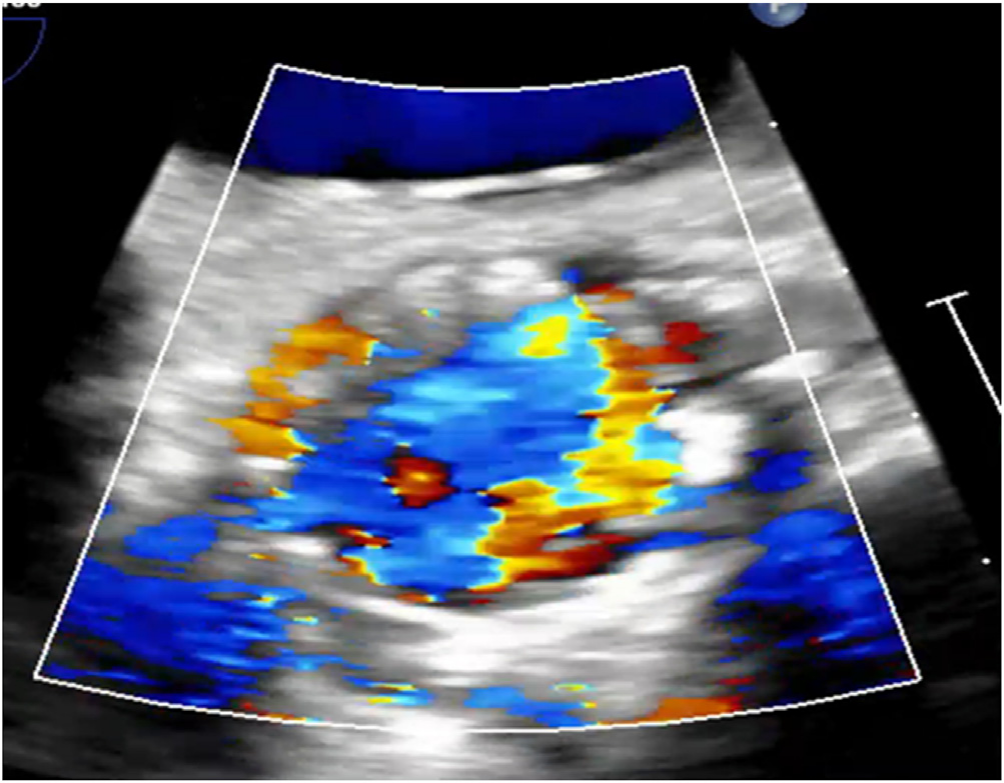
Transesophageal echocardiogram short axis of aortic valve view showing frozen leaflet causing severe AR. Abbreviation: AR, aortic regurgitation.

**Figure 2:**
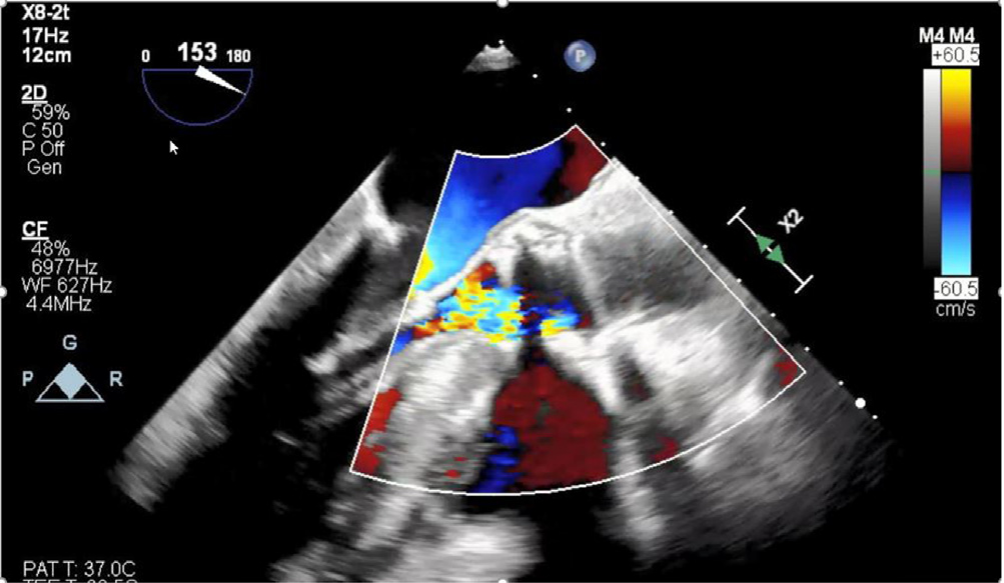
Transesophageal echocardiogram long axis view showing frozen leaflet causing severe AR. Abbreviation: AR, aortic regurgitation.

**Figure 3:**
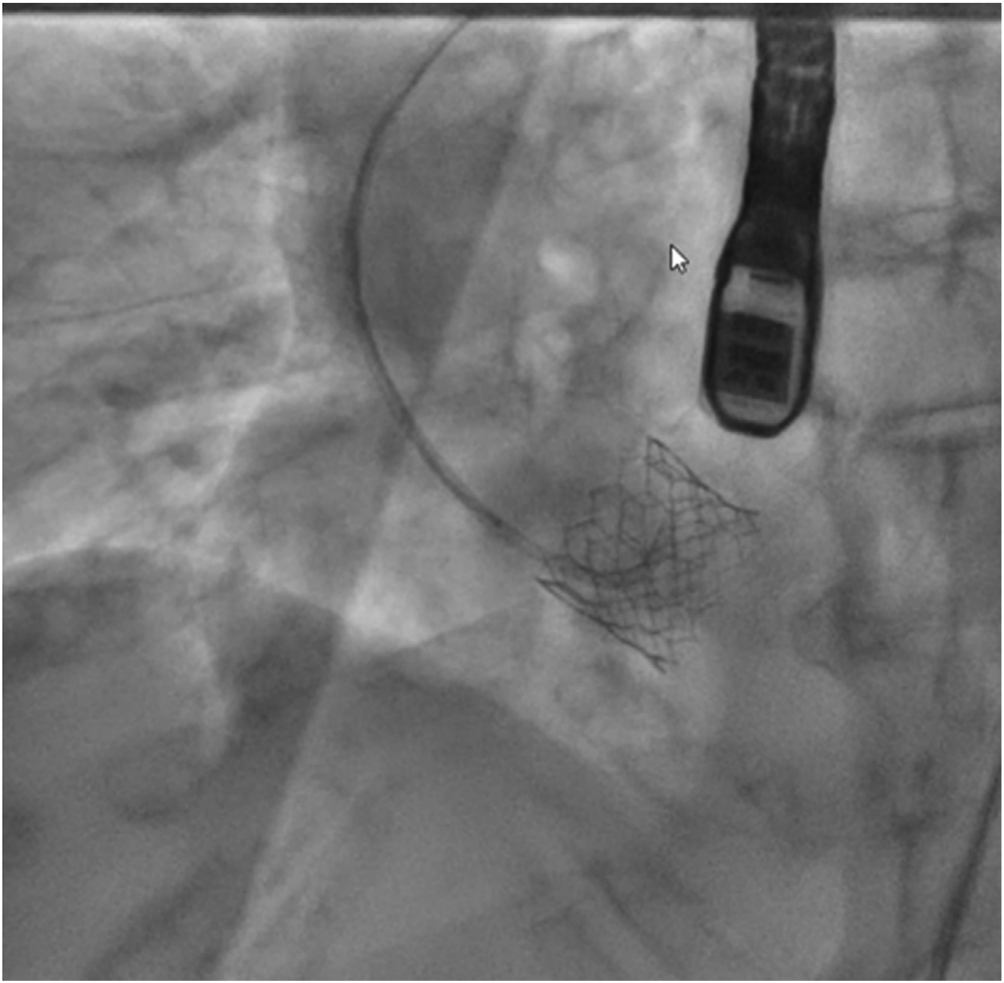
Fluoroscopy image showing JR4 catheter used to translocate the leaflet. Abbreviation: JR4, Judkins Right.

**Figure 4:**
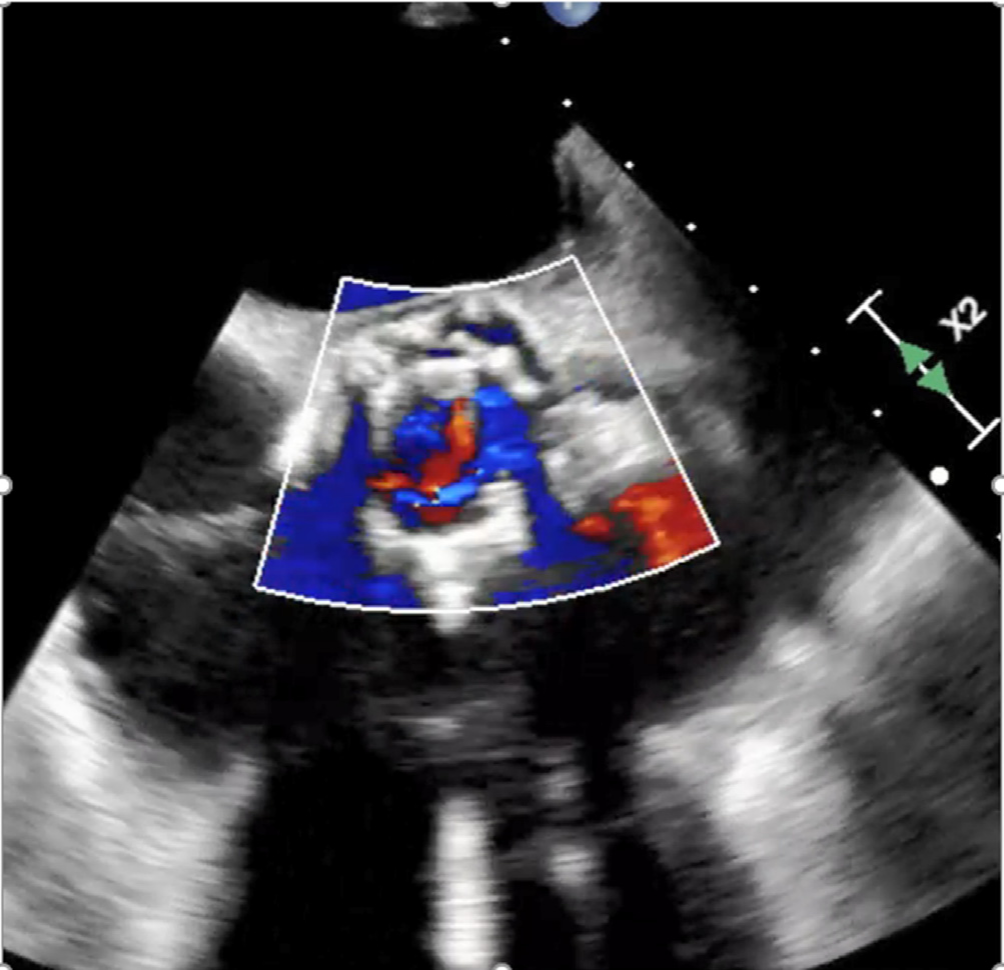
Transesophageal echocardiogram short axis of aortic valve showing resolution of aortic regurgitation.

This case underscores that an easy mechanical intervention with catheter manipulation for frozen leaflet translocation may obviate the need for further valve implantation and cost-effective in managing post transcatheter aortic valve replacement AR.

## Consent Statement

Consent was obtained from the patient for publication of this report and any accompanying images.

## Funding

The authors have no funding to report

## Disclosure Statement

The authors report no conflict of interest.
