# Supplementary figures and images for: An Easy Way to Solve the Stuck Leaflet Causing Aortic Regurgitation Following Transcatheter Aortic Valve Replacement
Source: Struct Heart. 2025 Jul 5;9(10):100697. doi: 10.1016/j.shj.2025.100697 (PMC12455104; doi:10.1016/j.shj.2025.100697)

## Slide 1
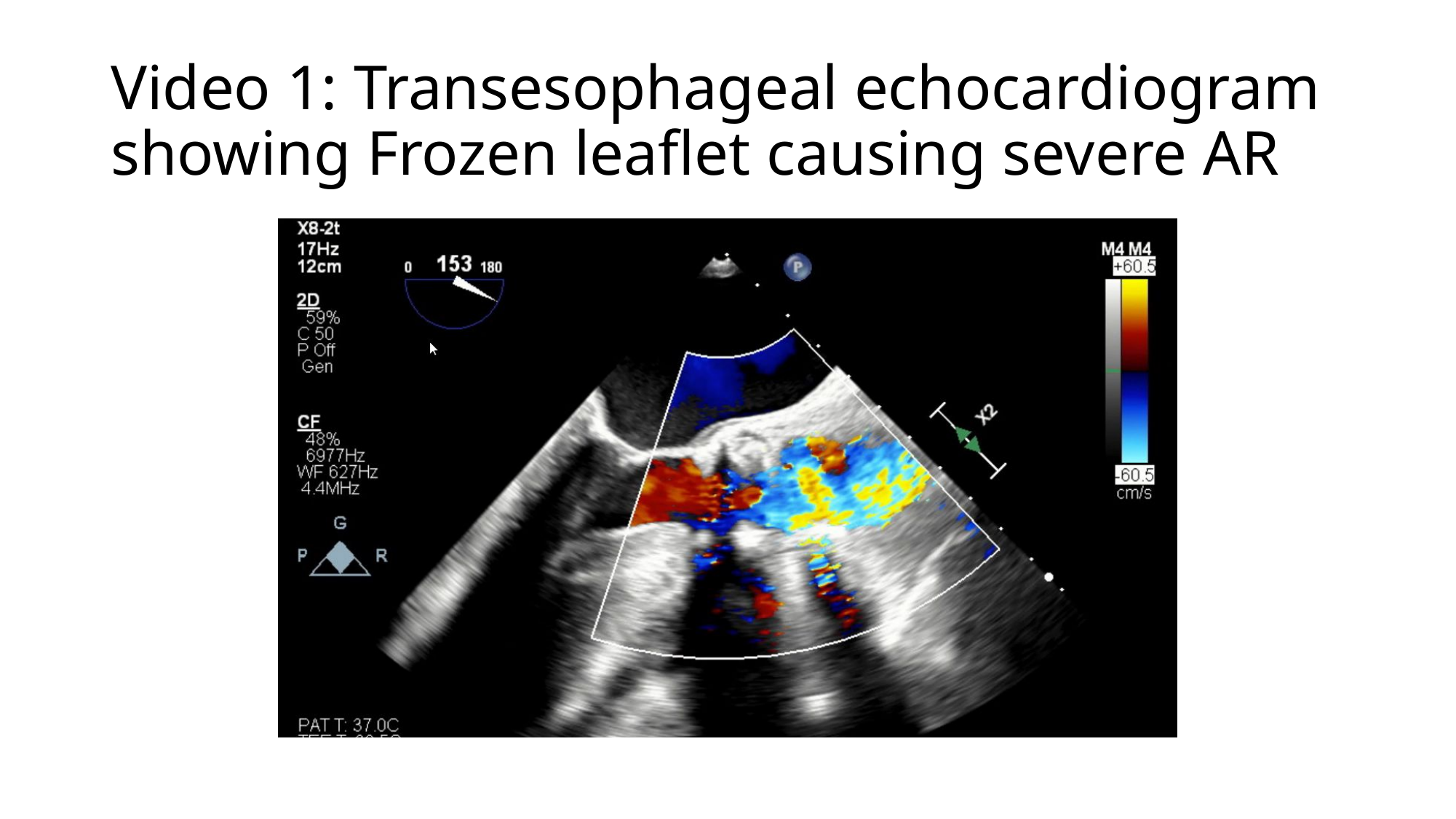

# Video 1: Transesophageal echocardiogram showing Frozen leaflet causing severe AR

Supplement: Supplementary file 1 — Supplementary Data [file mmc1.pptx]
